# Excited-State Charge Transfer Coupling from Quasiparticle Energy Density Functional Theory

**DOI:** 10.1021/acs.jpclett.4c00850

**Published:** 2024-06-03

**Authors:** Kai-Yuan Kuan, Shu-Hao Yeh, Weitao Yang, Chao-Ping Hsu

**Affiliations:** † Institute of Chemistry, Academia Sinica, 128 Academia Road, Section 2, Nankang District, Taipei 11529, Taiwan; ‡ Department of Chemistry, National Taiwan University, 1 Roosevelt Rd, Section 4, Da’an District, Taipei City 10617, Taiwan; ¶ Department of Chemistry, Duke University, Durham, North Carolina 27708, United States; § Division of Physics, National Center for Theoretical Sciences, 1 Roosevelt Road, Section 4, Taipei City 10617, Taiwan

## Abstract

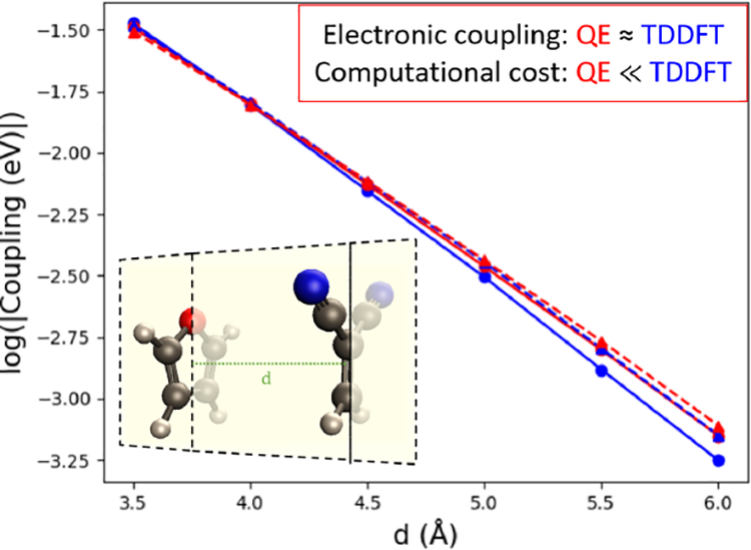

The recently developed Quasiparticle Energy (QE) scheme, based on a DFT calculation with one more (or less) electron, offers a good description of excitation energies, even with charge transfer characters. In this work, QE is further extended to calculate electron transfer (ET) couplings involving two excited states. We tested it with a donor–acceptor complex, consisting of a furan and a 1,1-dicyanoethylene (DCNE), in which two low lying charge transfer and local excitation states are involved. With generalized Mülliken-Hush and fragment charge-difference schemes, couplings from the QE approach generally agree well with those obtained from TDDFT, except that QE couplings exhibit better exponential distance dependence. Couplings from half-energy gaps with an external field are also calculated and reported. Our results show that the QE scheme is robust in calculating ET couplings with greatly reduced computational time.

Intermolecular electron transfer (ET) is a fundamental process in many optoelectronic, and biological systems.^1−7^ To gain insights into mechanisms of these processes, it is important to estimate their rates. In the weak coupling limit, the ET rate can be described by the Marcus theory, which is based on Fermi’s golden rule:^8,9^
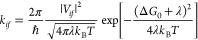
1where *V*_*if*_ is the electronic coupling factor describing the transition between the initial (*i*) and final (*f*) states, λ is the reorganization energy, and Δ*G*_0_ is the standard free energy difference between *i* and *f*. The expression of eq 1 involves a classical treatment for vibrations affecting state energies, whereas quantum treatment for high-frequency progression has also been developed. From eq 1, it is seen that the ET rate is proportional to the squared amplitude of electronic couplings, *V*_*if*_, and an accurate description of these couplings is needed for many physical processes, such as singlet fission,^10^ triplet energy transfer,^11^ or dynamical systems.^12^

Near the crossing of the initial and final electronic states, the Born–Oppenheimer approximation breaks down, and diabatic states that diagonalize the nuclear momentum operator can be proper bases for the ET process. However, such strictly defined states are overdetermined in most systems.^13^ As a result, calculation of coupling relies on the definition of diabatic states.^9^ For ET problems involving excited states, a linear combination of excited states of the donor–acceptor system can be taken, such that the dipole moment or charge separation of the system is maximized, leading to Generalized Mülliken-Hush (GMH)^14^ and fragment charge difference (FCD)^15^ approaches, respectively. Both two-state and multistate approaches have been developed.^16−18^ In general, both GMH and FCD are convenient and computationally efficient, and they are to some extent, quite robust to the quality of the Hamiltonian that generates the excited state.^19,20^ Nevertheless, in order to properly describe the two diabatic states involved in transfer processes, an affordable and reliable *ab initio* method for molecular excited-state properties is still required.

Conventionally, exited-state properties of an *N*-electron molecule are obtained via direct calculation of the *N*-electron system using time-dependent methods (e.g., linear-response TDDFT),^21,22^ coupled-cluster (CC) theory,^23,24^ and a Bethe-Salpeter equation incorporated into screened Coulomb interaction^25^ and quasi-particle energies (QE) from the GW method (GW-BSE).^26−30^ However, among those treatments, both the CC theory and GW-BSE are computationally demanding when large systems are considered. TDDFT, on the other hand, provides efficient access to large systems, but with typically used exchange-correlation functionals, it describes charge transfer (CT) and Rydberg excitations poorly.^31^

Excitation energies based on DFT can also be obtained from a computational process that does not conserve particle numbers. Starting from the ground state of an *N*-electron system, excited-state energies of the corresponding (*N* ± 2)-electron systems can be captured with the particle–particle random phase approximation (pp-RPA).^16^ pp-RPA is the simplest approximation of the paring field TDDFT,^32^ and accurately describes valence, double, Rydberg, CT excitations, and conical intersections^16,33−35^ and has been developed into a very efficient computational approach with active space truncation.^35,36^

Parallel to pp-RPA, starting from the ground state of an *N*-electron system, excited stated energies of the corresponding (*N* ± 1)- electron system can be captured with the quasiparticle energy (QE-DFT) approach.^37^ QE-DFT was developed by the Yang group^38,39^ and the Bartlett group.^40^ It uses orbital energies of an *N*-electron system ground state as an approximation of quasiparticle energies and by extension, of excited state energies of the corresponding (*N* – 1)-electron system with occupied orbitals and of the corresponding (*N* + 1)-electron system with virtual orbitals. Note that HOMO and LUMO orbital energies in DFT have proven to be chemical potentials of electron removal and addition, respectively.^41^ The meaning of chemical potentials of HOMO and LUMO energies and linearity conditions for ground state energies of fractional particle numbers^42,43^ rigorously establish HOMO and LUMO energies as approximations of corresponding quasiparticle energies from a given DFT approximation. The related physical meaning of the remaining orbital energies has not been established, but is supported with extensive numerical conditions. The QE-DFT approach accurately describes valence excitations with commonly used DFT approximation, and Rydberg and CT excitations^37^ with the localized orbital scaling correction (LOSC)^44,45^ was developed to eliminate the delocalization error in DFT.^46,47^ The QE-DFT is the simplest approach to excited states — as it only requires a ground state DFT calculation. Corresponding use of quasiparticle energies for excitation energy calculations has also been developed with accurate results, based on a many-electron Green’s function GW approach.^48^

A major remaining issue is the delocalization error in commonly used density functional approximations (DFAs), which would cause significant errors in predicting orbital energies. Implementation of double hybrid^49−53^ or long-range corrected^54−60^ functionals is a potential solution for tackling delocalization errors. Previously, we developed LOSC approximations to minimize delocalization errors in DFA.^46,47^ LOSC-corrected QE effectively describes CT energies, particularly at the dissociation limit. Implementation of LOSC in the QE approximation accurately describes excitation properties such as electronic densities,^61^ polarizabilities,^62^ and CT excitation energies at the dissociation limit.^63^

In the present work, for the first time, we apply QE-DFT, including the LOSC approximation, to calculate electronic couplings. In the following section, the theory behind calculation of electronic coupling is explained. From our testing results and discussions, we conclude that QE-DFT can be a cost-effective, useful approach for charge-transfer couplings.

## Excitation Energies from QE Methods

The conventional definition of QE is the energy cost to create an electron or a hole in an interacting system. Quasiparticle/quasihole energies ω^±^(*N*) of an *N*-electron system can be expressed in terms of *E*_0_(*N*), the ground-state of the *N*-electron system and *E*_*m*_(*N* + 1) or (*E*_*n*_(*N* – 1)), the ground or excited-state energies of the corresponding (*N* + 1) or (*N* – 1))-electron system, as
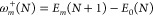
2and
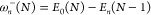
3where the plus/minus sign in the superscript of ω indicates addition/removal of an electron to create a particle/hole from the *N*-electron ground state system.^38,39^ With the QE-DFT approach, ε_*m*_(*N* – 1), generalized Kohn–Sham energies of occupied orbitals in the (*N* – 1)-electron ground state approximate ω_*m*_^+^(*N* – 1). Note that ε_LUMO_(*N* – 1), the LUMO energy for the (*N* – 1) system, connects to *E*_0_(*N*), the *N*-electron ground state energy. The excitation energy, Δ*E*_*m*_(*N*) becomes
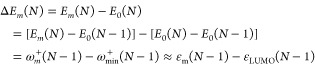
4

Similarly, excitation energy Δ*E*_*n*_(*N*) in the *N*-electron system can be obtained from ground state of the (*N* + 1) system:
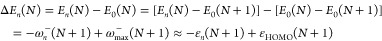
5Therefore, eq 4 and eq 5 show that excitation energy can be approximated simply as the energy difference between orbital energy of LUMO/HOMO and one of the corresponding occupied orbitals in the (*N* – 1) (or (*N* + 1))-electron system.^38,39^

## Conventional Description of Electronic Couplings

To obtain charge-localized states from eigenstates of the Schrödinger equation, GMH^14^ and FCD^15^ are two commonly used schemes that can be easily computed from entry-level excited-state methods. In GMH, diabatic states are eigenstates of a dipole operator, a choice that maximizes the dipole moment difference between the two states. The ET coupling, *V*_*mn*_^GMH^, derived from eigenstates |*m*⟩ and |*n*⟩ is written as
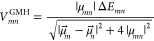
6where the Δ*E*_*mn*_ is the energy difference between the two states, |μ_*mn*_| is the net transition dipole moment between the two states, and μ⃗_*m*_ (≡ μ⃗_*mm*_) and μ⃗_*n*_ (≡ μ⃗_*nn*_) are permanent dipoles, all from eigenstate descriptions. Components (consider the *x* direction as an example) of these dipole moments can be calculated from
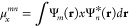
7where Ψ_*m*_(**r**) and Ψ_*n*_(**r**) represent excited states |*m*⟩ and |*n*⟩. The other two directions can be made in an analogous way. The net transition dipole becomes
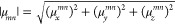
8

In FCD, user-defined donor and acceptor fragments are employed to define a 2 × 2 donor–acceptor charge difference matrix, **Δq** with its matrix element Δ*q*_*mn*_, between eigenstates |*m*⟩ and |*n*⟩:
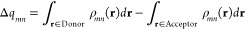
9where diagonal elements Δ*q*_*mm*_ (≡ Δ*q*_*m*_) are calculated from the diagonal one-particle density, ρ_*mm*_(**r**), and the off-diagonal Δ*q*_*mn*_ is the corresponding quantity from the transition density, ρ_*mn*_(**r**) of the two states, |*m*⟩ and |*n*⟩,
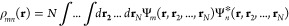
10where Ψ_*m*_ is the many-electronic wave function of state |*m*⟩, and *N* is the number of electrons in the system. By requiring the maximum charge separation difference in diabatic states, the FCD coupling, *V*_*mn*_^FCD^, derived from eigenstates |*m*⟩ and |*n*⟩ is then^64^
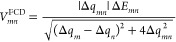
11

## Electronic Couplings between Two Excited States under the QE-DFT Scheme

In TDDFT, the one-particle density (*m* = *n*) and the transition density (*m* ≠ *n*) can be obtained by projecting the corresponding transition eigenvector associated with the pole in the linear response to a single excitation model.^65^ The QE-DFT approach describes an excited state as a one-particle transition from one to another orbital of the reference quasiparticle/quasihole orbital. For two excited states, (|*a*⟩ and |*b*⟩), the excitation energies (*E*_*a*_ and *E*_*b*_) expressed in the (*N* – 1)-electron system quasiparticle energies are
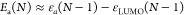
12
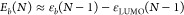
13Here we use 1-electron orbital energies for excited state energies. Thus, the indices, *a* and *b*, can be used to specify *both* the desired states *and* their corresponding orbitals. The energy difference between |*a*⟩ and |*b*⟩ becomes
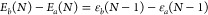
14

Likewise, for two excited states (|*i*⟩ and |*j*⟩) describable in the (*N* + 1)-electron quasihole systems, the excitation energies (*E*_*i*_ and *E*_*j*_) are
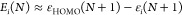
15
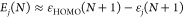
16Their energy difference becomes
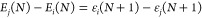
17Due to the similarity of formulations between the (*N* – 1)- and (*N* + 1)-electron systems, we use the (*N* + 1)-electron system as an example for the following discussions. The same idea applies to the (*N* – 1)-electron system.

Since the energy difference of two excited states can be approximated simply as the energy difference of two quasiparticle/quasihole orbitals, it can be hypothesized that other transition properties, such as transition dipoles or electronic couplings, can also be approximated using orbitals in these systems. By replacing excited states, Ψ_*m*_ and Ψ_*n*_, with their single configurations involving removal of an electron from an occupied orbital in the (*N* + 1)-electron system, φ_*i*_^*N*+1^ and φ_*j*_^*N*+1^, the one-particle density or transition density ρ_*ij*_(**r**) in eq 10 is
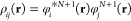
18

Following eq 9 the charge difference matrix **Δq**^QE^ is then,
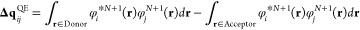
19The first term in eq 19 can be calculated as
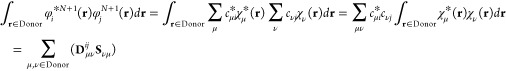
20and a generalization to the second term is straightforward. For the diagonal element Δ*q*_*ii*_ (≡ Δ*q*_*i*_) and Δ*q*_*jj*_ (≡ Δ*q*_*j*_), the density of the background reference state ρ_(*N*+1)_ contributes constant values in the Mülliken population, regardless of the state indexes *i* or *j*. Therefore, Δ*q*_*i*_ and Δ*q*_*j*_ are shifted by an identical constant value when only φ_*i*_ or φ_*j*_ is considered in Δ*q*.

The GMH scheme has an expression similar to that of FCD. The difference is to replace dipole moment (μ_*m*_) and transition dipole (μ_*mn*_) of the entire system with the charge and transition density of the user-defined fragment, respectively. Both quantities can be obtained from a multiple-density matrix with dipole matrices of atomic bases (eq 21).
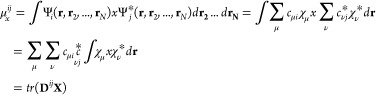
21where **X** is the operator of vector (*x*, *y*, *z*) in AO basis, and μ_*x*_^*ij*^ represents the permanent dipole of orbital *i* or *j* (*i* = *j*), or transition dipole between orbital *i* and *j* (*i* ≠ *j*) on the *x*-axis. The magnitude of μ_*ij*_ to be applied in eq 6 is the square-root of the squared sum of dipole matrices in each direction with the other two directions made in an equivalent way (eq 7).

Note that the Δ*E*_*mn*_ for calculating GMH (eq 6) and FCD (eq 11) couplings utilizes the nonspin-purified Scheme eq 2 because the way to obtain the corresponding “spin-purified” wave function for calculating the dipole moments (μ^*mn*^) in GMH or the partial charge (Δ*q*_*mn*_) in FCD still remains unclear.

## Electronic Couplings from an External Electric Field Scan

In the two-state model, an electron transfer event can be expressed as a transition from the initial to the final state of the system, in which the Hamiltonian can be written in two forms:
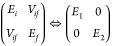
22where the basis of the left side is the charge-localized, diabatic state, with its energies *E*_*i*_ and *E*_*f*_, and the coupling between the two states *V*_*if*_. The right side is expressed under the eigenstates of the Hamiltonian, with eigenvalues *E*_1_ and *E*_2_, which can be written as
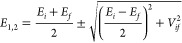
23In a resonant condition (*E*_*i*_ = *E*_*f*_), the energy gap between two adiabatic states is simply twice that of the coupling (*E*_1_ – *E*_2_ = 2*V*_*if*_). Such a condition can be achieved by tuning the external electric field. For two adiabatic states in the same symmetry group, the avoid-crossing region shows up when energies of the two states approach one another upon external perturbation, such as by electric fields. At a certain point, the energy gap between the two states reaches the minimum and the corresponding diabatic states become degenerate. At this point, coupling can be obtained by half of the adiabatic energy gap.^66,67^

## Methods

### Choice of Basis Sets

**Figure 1:**
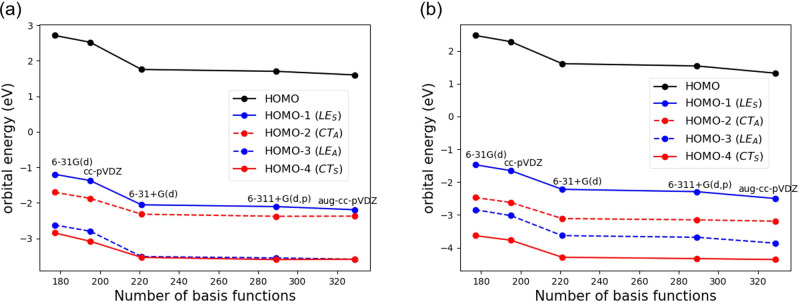
Orbital energies of the (*N* + 1)-electron system of the furan-DCNE complex using the BLYP method with different basis sets at (a) *d* = 3.5 Å and (b) *d* = 6.0 Å.

Since the accuracy of QE methods on the (*N* + 1)-electron system may be sensitive to the choice of basis sets, stability checks on different basis sets were performed. Figure 1) shows energies of the five orbitals shown in Figure 6 calculated using a BLYP functional with different basis sets. Although energies change a lot for small basis sets, they reach convergence for larger ones, as long as diffusion functions are included (6-31+G*, 6-311+G**, aug-cc-pVDZ). As a result, we chose the 6-31+G* basis set for the following studies, unless specified.

### Electronic Couplings from External Electric Fields

As an alternative approach to validate calculated couplings for this system, an energy gap scheme to find the minimum energy gap between two states of interest by changing external electric fields was also applied for comparison with our methods. In finding the minimum excitation gap by changing external electric fields, two point charges separated by 20,000 Å centered at the origin were placed along the *z*-axis.^62^ The scan was repeated, but with smaller increments of point charge strength until orbital energies (in hartree) of the two states converged at three significant figures at the minumum gap and the adjacent two points (see the Supporting Information for details).

### Other Computational Details

Structures, energies, and electronic couplings of GMH and FCD from TDDFT were employed using Q-Chem 6.0.^68^ Orbitals were visualized on IQmol v3.0. LOSC-related calculations utilized the package, QM4D, developed by Yang’s group, with LOSC2 version.^61^

### State Assignments

**Figure 2:**
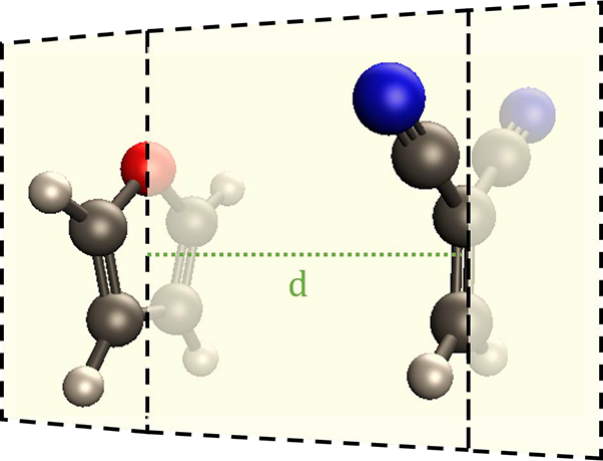
Stacked furan-DCNE complex and the σ-plane used to determine state and orbital symmetry in this work. The intermolecular distance, *d*, indicates the distance between the center of two fragments, as indicated by the green dashed line.

**Figure 3:**
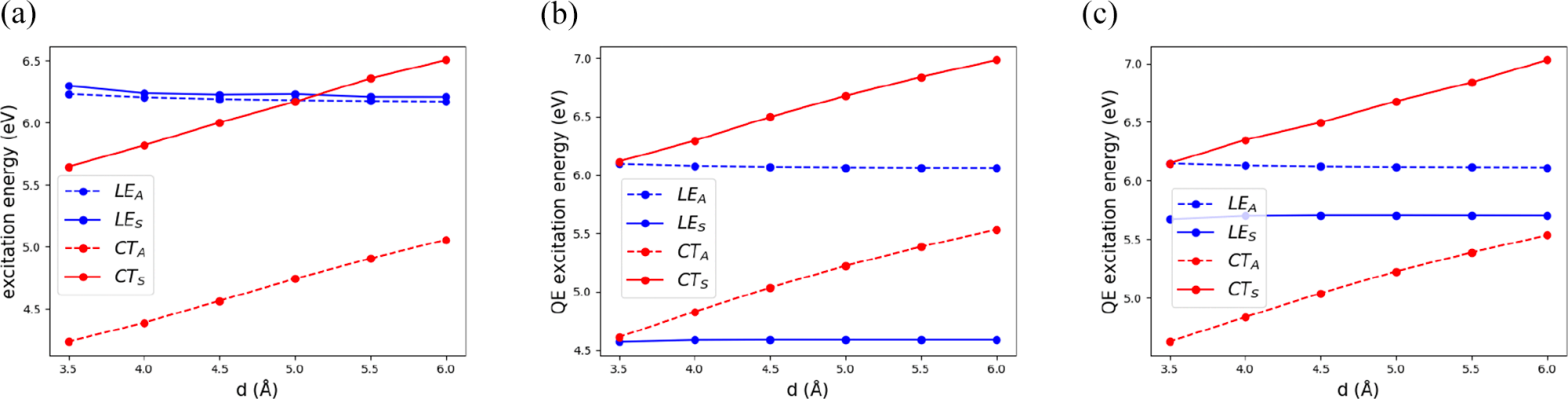
(a) TD excitation energy of the four states of interest, (b) excitation energies calculated using the QE method with eq 2, and (c) excitation energies calculated using the QE method with a spin purification process from eq 24. All calculations are in ωB97xD/6-31+G* method.

We selected a furan and 1,1-dicyanoethylene (DCNE) face-to-face complex as our model system (Figure 2). This is reported to have a low-lying CT excitation at short distances.^69^ We chose the first four excitations (denoted as LE1, LE2, CT1, and CT2 in their work) for our study. Figure 3a shows excitation energies calculated by the TD-ωB97xD/6-31+G* method for different intermolecular distances (*d*) from 3.5 to 6.0 Å. Both CT excitations show strong distance dependence and the order of states changes at longer distances for higher lying CT with LE excitations. This is consistent with their reported excitation energies using 6-31G* as the basis set. To avoid confusion on the order of states at different *d*s, we relabel these excitations according to the σ plane of the *C*_*s*_ symmetry of the complex. As a result, the original labeling CT1, CT2, LE1, and LE2 becomes CT_*A*_, CT_*S*_, LE_*A*_, and LE_*S*_, respectively, in this work, with the subscript *A* indicating antisymmetric and *S* indicating symmetric.

**Figure 4:**
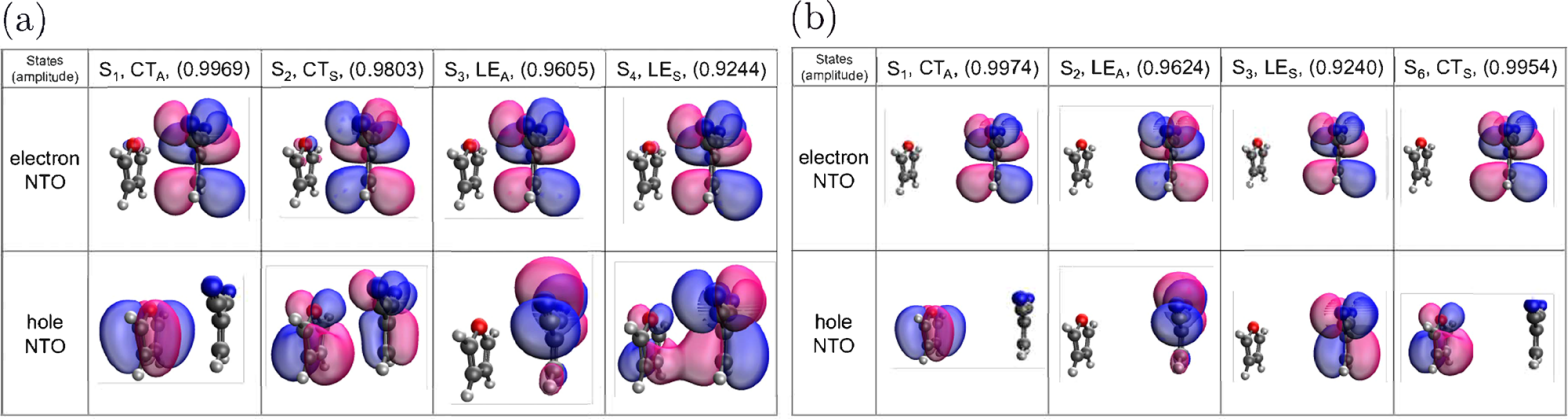
Natural transition orbital (NTO) pairs of CT_*A*_, CT_*S*_, LE_*A*_, and LE_*S*_ at distances (a) 3.5 Å and (b) 6.0 Å, calculated by TD-ωB97xD. Numbers in parentheses indicate amplitudes of the major transitions.

From a direct TDDFT calculation, the natural transition orbitals (NTOs) of the four excitations are shown in Figure 4 for *d* = 3.5 and 6.0 Å using the TD-ωB97xD/6-31+G* method. The electron-NTOs are very similar for all these excitations, indicating that a π* orbital of DCNE, and the hole-NTOs can be one of the two π orbitals of furan (CT states), or a π or a nonbonding (*n*) orbital of DCNE. At *d* = 3.5 Å, CT_*S*_ renders a slight mixing with LE_*S*_ and such mixing is absent for *d* = 6.0 Å, indicating distance-dependent coupling between the two states.

**Figure 5:**
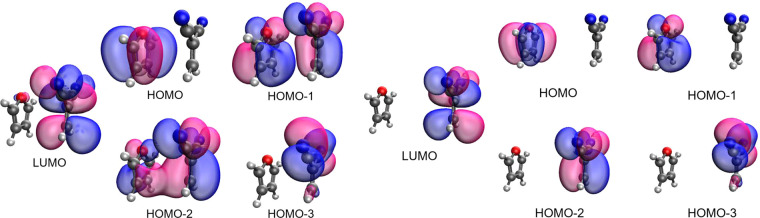
Canonical orbitals calculated by ωB97xD/6-31+G* of the *N*-electron system from HOMO–3 to LUMO for (a) *d* = 3.5 Å and (b) *d* = 6.0 Å.

**Figure 6:**
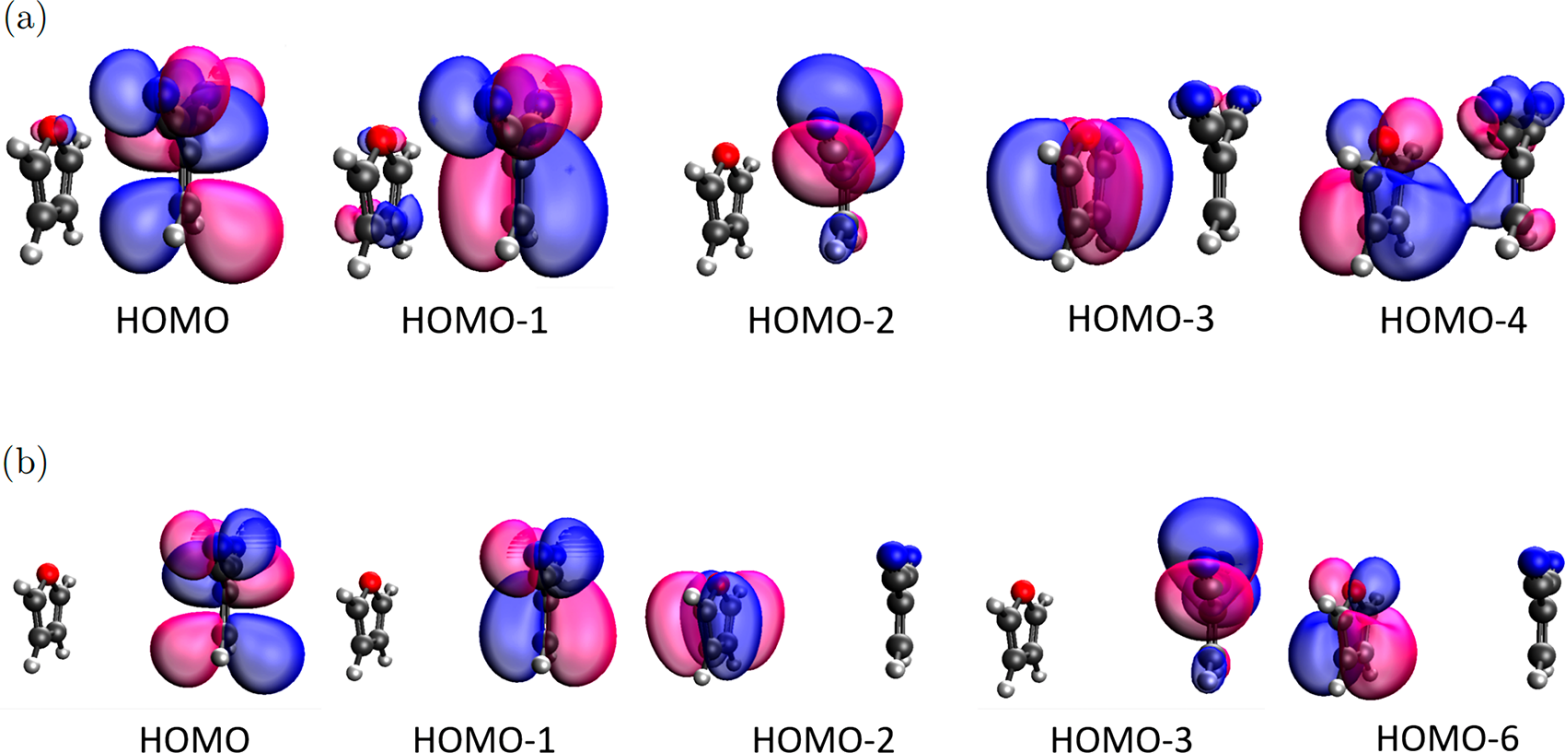
Alpha orbitals of the (*N* + 1)-electron system at (a) *d* = 3.5 Å and (b) *d* = 6.0 Å, calculated by ωB97xD.

Excitation of an *N*-electron system can be approximated from QE either with (*N* – 1)- or (*N* + 1)-electron reference system approaches.^38,39^ Conventionally, an approach from (*N* – 1)-electron systems has advantages over that from (*N* + 1)-electron systems: virtual orbitals of the (*N* – 1)-electron system are more likely to be bounded and are less demanding in regard to choice of the basis set. However, comparing NTOs (Figure 4) with canonical orbitals (Figure 5), the major single-excitation component of the transition from one of the occupied orbitals to the LUMO can be deduced. This can also be found from the close-to-one amplitude of single-excitation (Figure 4). This implies that the (*N* + 1)-electron system is more appropriate for describing these excitations. Figure 6 shows the occupied orbitals of the (*N* + 1)-electron system that correspond to those in Figure 5. As expected, the lower occupied orbitals in the (*N* + 1)-electron system show one-to-one correspondence with hole NTOs, and the HOMO (LUMO in the *N*-electron system) corresponds to electron NTOs for all four excitations. Therefore, the (*N* + 1)-electron system was used for the QE reference in the following discussion.

### Excitation Energies

QE methods with LOSC corrections can be applied to accurately calculate CT excitations.^63^ To begin, we calculated excitation energies for all four excited states. Figure 3 compares excitation energies calculated using the TD and QE methods (eq 5). Qualitatively, the two CT states depend strongly on the donor–acceptor distance, while neither LE state does. Quantitatively, excitation energies on CT_*A*_, CT_*S*_, and LE_*A*_ states calculated by the QE method (Figure 3b) are close to those calculated from TD (Figure 3a) with a small shift of 0.5 eV. This is consistent with previous work by Xie et al. where significant distance dependence on excitation energies was observed for CT states.^69^ The LE_*S*_ state exhibits a larger shift (∼2 eV). However, we note that the energy difference between the α and the corresponding β orbital describing the hole of the LE_*S*_ state is much larger than the other three. This implies that the energy changes upon spin-purification of LE_*S*_ state would be large.

For a closed-shell *N*-electron system, the reference from the (*N* + 1)-electron QE system is an open-shell doublet state. Note that removal of one electron from the (*N* + 1)-electron QE system produces a spin-mixed state (*E*_*↑↓*_), which can be regarded as approximately a half–half mixture of singlet and triplet states. Since the corresponding triplet state can be obtained by removing an electron from the corresponding β orbital, the pure singlet state can be calculated by a process called spin-purification^38,39,70^ by
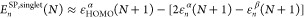
24

Figure 3c shows spin-purified excitation energies of the four states. It shows that spin-purification reduces the difference of energies calculated by the TD and QE methods of the LE_*S*_ state to ∼0.5 eV, whereas the other three states remain nearly unchanged. Although we are not saying that TD is more accurate than QE or vice versa, it still indicates that the QE, being a computationally simpler approach, provides good approximations for systems that are commonly described by TD approaches.

### Couplings in the GMH and FCD Schemes: Comparison between TD and QE Methods

As a low-cost approach, QE provides a very simple description for excitation with only a pair of orbitals. Here we test the performance of QE in electronic coupling of two excited states, employing GMH and FCD schemes. As described in eq 6 and eq 11, GMH requires matrix elements of dipoles, whereas FCD requires populations. Performance in GMH and FCD coupling relies on the quality of such of quantities from QE, which offers important insight for future application of QE.

**Figure 7:**
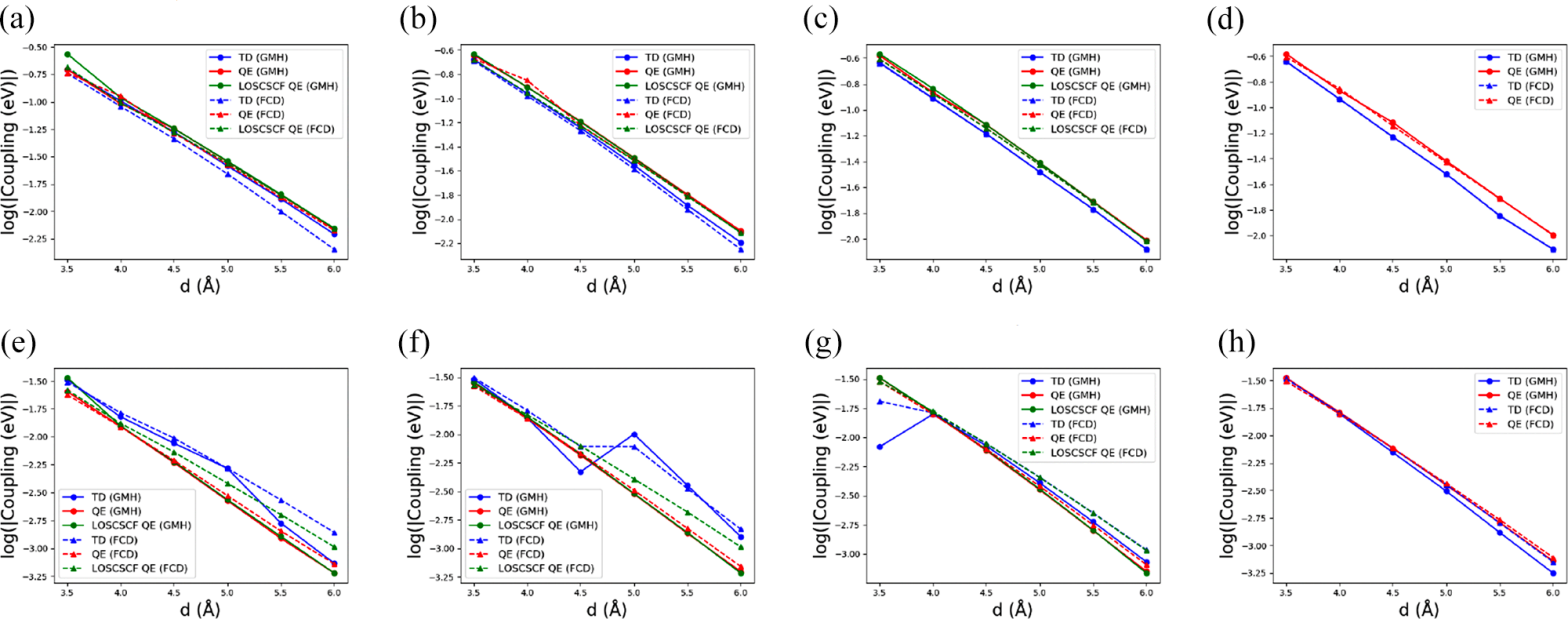
Couplings of LE_*S*_–CT_*S*_ calculated by (a) BLYP, (b) B3LYP, (c) CAMB3LYP, and (d) ωB97xD, and couplings of LE_*A*_–CT_*A*_ calculated by (e) BLYP, (f) B3LYP, (g) CAMB3LYP, and (h) ωB97xD in logarithmic scale. All basis sets are 6-31+G*.

Since ET coupling typically decays exponentially with donor–acceptor distance, coupling, *V*_*if*_, can be expressed as
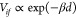
25where *d* is the donor–acceptor distance and β is the decay rate. The β is a useful quantity in determining the mechanism of electron (hole) transfer processes since it is sensitive to the nature of the donor–acceptor system and the tunneling medium.^71,72^Figure 7 shows FCD and GMH couplings between LE and CT excitations calculated by TD and QE methods. (The absolute value for each point is summarized in the Supporting Information.) LOSC-SCF was also employed for the QE method to reduce potential problems with delocalization errors. The present results show that these coupling values decay exponentially with donor–acceptor distance in clear linear fashion in semilog plots. The distance dependence is mostly independent of methods used. However, couplings calculated from TDDFT exhibit some off-linear points, whereas those calculated from QE approaches are mostly linear. This suggests that QE provides a more robust way of estimating the distance dependence than TDDFT.

For a more quantitative comparison, Table 1 summarizes the fitted decay rate (β) of each line in Figure 7. Two effects can be derived here. First, couplings calculated with a pure DFA functional (BLYP) exhibit a larger difference between TD and QE methods, whereas those calculated by range-separated hybrid functionals (CAMB3LYP or ωB97xD) are more consistent. This is supported by our previous study showing that using functionals with range-separated parameters can improve predictions of electron (hole) transfer couplings due to better descriptions of asymptotic potentials.^73^ Second, this better description in the asymptotic region was also shown by implementation of LOSC to correct delocalization errors in QE-DFT. We demonstrated previously that LOSC provides an accurate picture of the 1/R dependence of the benzene-TCNE complex on the first singlet CT excitation energy.^63^ This was achieved by minimizing the delocalization error in pure DFA functionals.^38,39^ However, such an error is not so obvious in this system because the QE LOSC-SCF values are very close to those in QE (without LOSC corrections). This is probably because furan is a better electron donor than benzene, such that orbitals are already highly localized in furan from DFA, implying that the delocalization error may be small.

**Table 1:** Decay Rate (β, Å^–1^) as in *V*_*if*_ ∝ exp(−β*d*) Calculated from GMH, and FCD of LE_*S*_–CT_*S*_ and LE_*A*_–CT_*A*_

scheme		GMH			FCD		
excitation pair	functional^a^	TD	QE	QE LOSC-SCF	TD	QE	QE LOSC-SCF
LE_*S*_–CT_*S*_	BLYP	1.38	1.34	1.41	1.48	1.35	1.33
	B3LYP	1.40	1.37	1.35	1.44	1.36	1.32
	CAMB3LYP	1.33	1.32	1.33	1.33	1.29	1.30
	ωB97xD	1.36	1.31	–b	1.36	1.29	–b

LE_*A*_–CT_*A*_	BLYP	1.49^c^	1.51	1.59	1.23	1.41	1.28
	B3LYP	1.10^c^	1.53	1.54	1.14^c^	1.46	1.31
	CAMB3LYP	1.47^d^	1.53	1.54	1.36^d^	1.45	1.34
	ωB97xD	1.54	1.53	–b	1.49	1.48	–b

a All basis sets are 6-31+G*.

b ωB97xD functional is not available in current version of QM4D package for LOSC calculation.

c Data deviate from a linear trend.

d Data for *d* = 3.5 Å was not included.

### Electronic Couplings from an External Electric Field Scan

**Figure 8:**
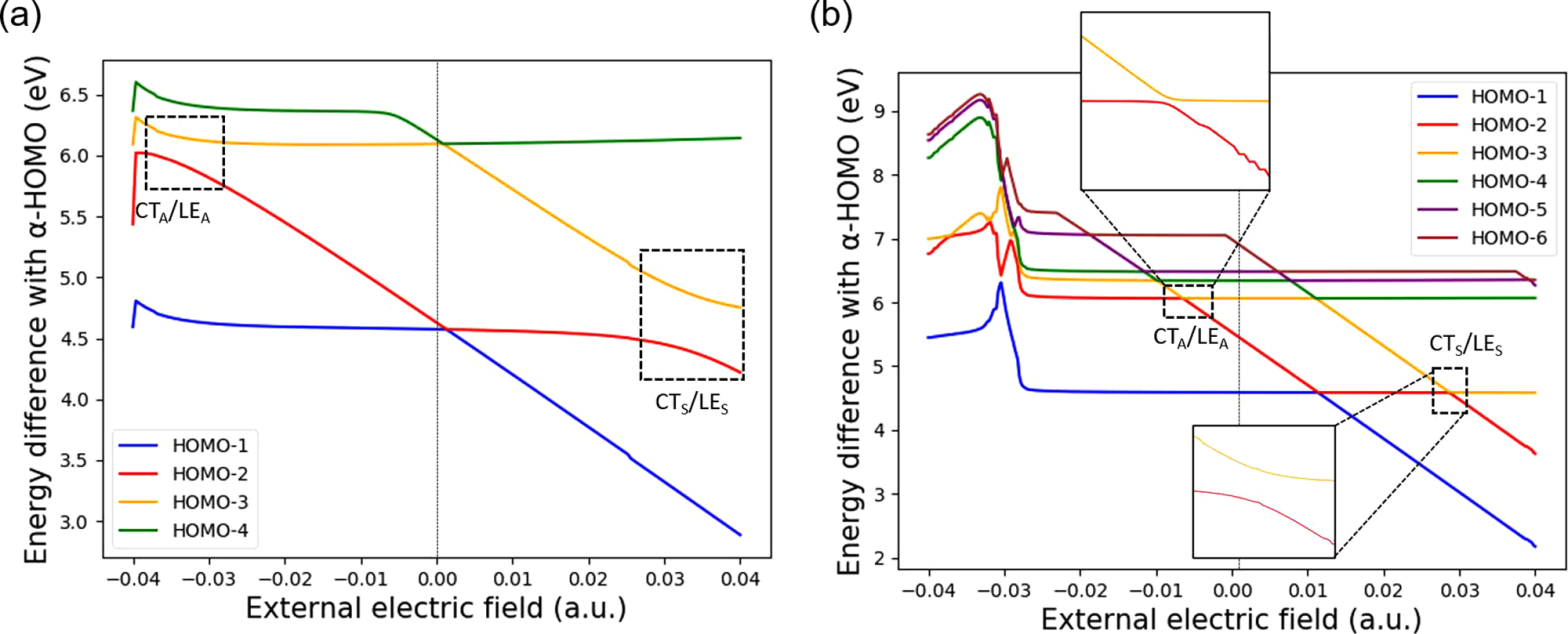
Orbital energy differences with respect to the HOMO versus external electric field strength calculated with the ωB97xD functional for (a) *d* = 3.5 Å and (b) *d* = 6.0 Å. The dashed boxes refer to “avoid-crossing” regions between CT_*S*_/LE_*S*_, and between CT_*A*_/LE_*A*_.

Since coupling can also be calculated using the electric field (EF) method introduced in the Theory section, it is employed to calculate the coupling as an additional comparison with the TD and the newly proposed QE method in this work. Figure 8 shows spectra of orbital energies by scanning the external electric field from −0.04 to +0.04 au This was achieved by placing two identical charges with strength ranging from −2.0 × 10^6^ to +2.0 × 10^6^ au located at ±10, 000 Å respectively on the axis connecting the center of the two molecular fragments. The two coupling values discussed in this work can be calculated by half of the minimal gap in the avoid-crossing region highlighted by dashed boxes in Figure 8.

**Figure 9:**
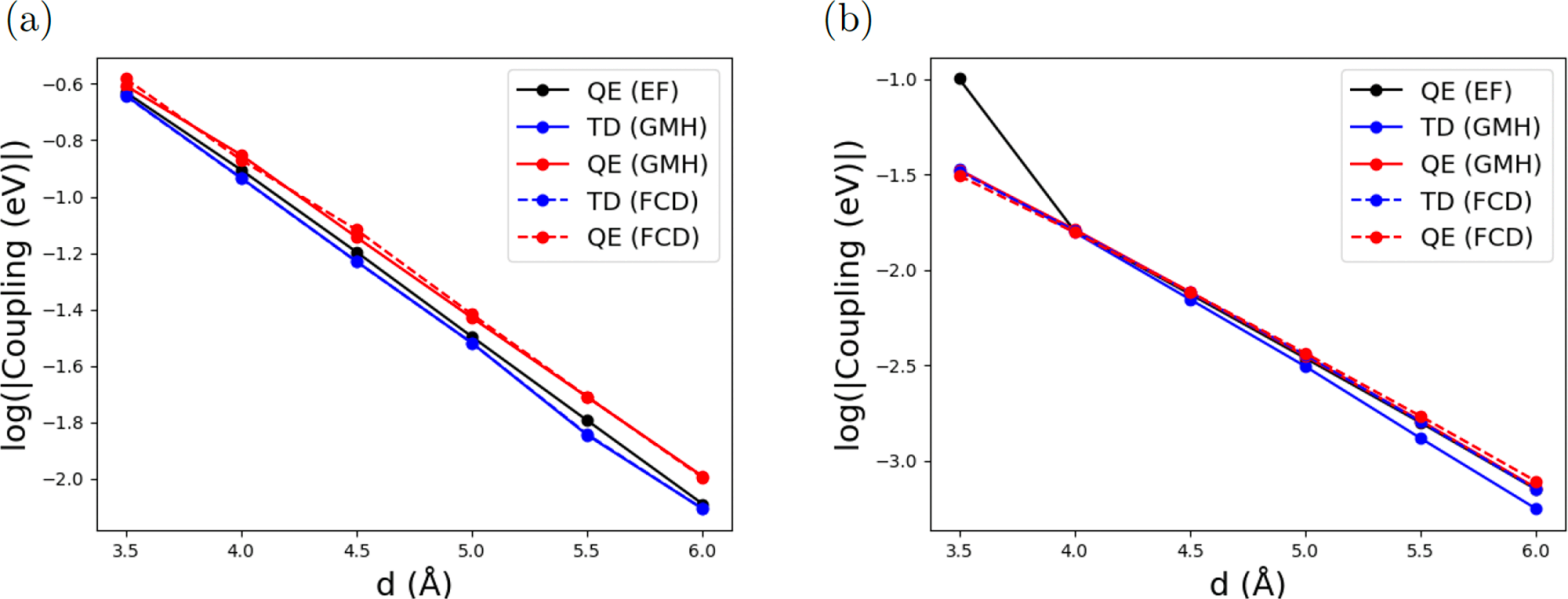
Electronic couplings calculated with a minimal orbital energy gap (QE (EF), black) and comparison with GMH and FCD schemes for (a) LE_*S*_ – CT_*S*_, and (b) LE_*A*_ – CT_*A*_ calculated by ωB97xD/6-31+G* method.

To search for the minimal gap between two orbitals, an incremental scan of the electric field strength around the avoid-crossing region was performed. This method results in the upper bound of the true coupling. Table 2 summarizes absolute couplings of LE_*S*_–CT_*S*_ and LE_*A*_–CT_*A*_ with the relative error attached, and Figure 9 compares the TD and QE methods. The *V*_EF_ fits well with TD and QE results with the β being 1.31 and 1.56 Å^–1^ respectively. This result supports the validity of the converged β calculated using TD with range-separated hybrid functionals and the QE approach.

**Table 2:** Couplings Calculated Using the Electric Field Method (*V*_EF_) for LE_*S*_–CT_*S*_ and LE_*A*_–CT_*A*_ Using ωB97xD

*d* (Å)	LE_*S*_ – CT_*S*_	LE_*A*_ – CT_*A*_
3.5	0.234	0.100
4	0.124	0.0160
4.5	0.0637	7.47 × 10^–3^
5	0.0319	3.45 × 10^–3^
5.5	0.0161	1.58 × 10^–3^
6	8.13 × 10^–3^	7.08 × 10^–4^

β (Å^–1^)	1.31	1.56^a^

a Excluding *d* = 3.5 Å.

General agreement in the coupling derived from GMH/FCD, with that from EF, is confirmation of the Condon approximation.^74^ Under perfect resonance, GMH and FCD expression would produce half the energy gap (eq 23 with *E*_*i*_ = *E*_*f*_); thus, coupling derived from EF can be viewed as both GMH and FCD coupling, except for an applied electric field. Since the electronic coupling derived from our original GMH and FCD schemes is obtained without an electric field applied, our results (in Table 2) indicate that the electric field, as an external parameter, does not change the electronic coupling value much.

We note that the QE approach is very similar to the Koopmans theorem approach for ground-state ET coupling.^75^ For a pair of closed-shell molecules, ET (or hole transfer, HT) coupling can be calculated from half the energy gap for two open-shell states with an additional electron (or hole). However, in such open-shell situations, radical anionic (or cationic) states are highly nondynamically correlated. Good ways to cope with such nondynamical correlation include adopting the closed-shell *N* – 1 (or *N* + 1, with *N* being the number of electrons in the target radical state as a reference state, with a Hartree–Fock Koopmans theorem (HF-KT), or a equation-of-motion coupled cluster treatment.^76^ With KT, the orbital energies of the usually neutral, closed shell, bimolecular system were obtained, and the energy gap of targeted states is the gap of two lowest unoccupied molecular orbitals (or two highest occupied molecular orbitals), from which ET (or HT) coupling can be derived. Such similarity of HF-KT to QE implies that a more general perspective or theoretical basis may exist for QE, a direction we aim to develop further.

### Computation Time

**Figure 10:**
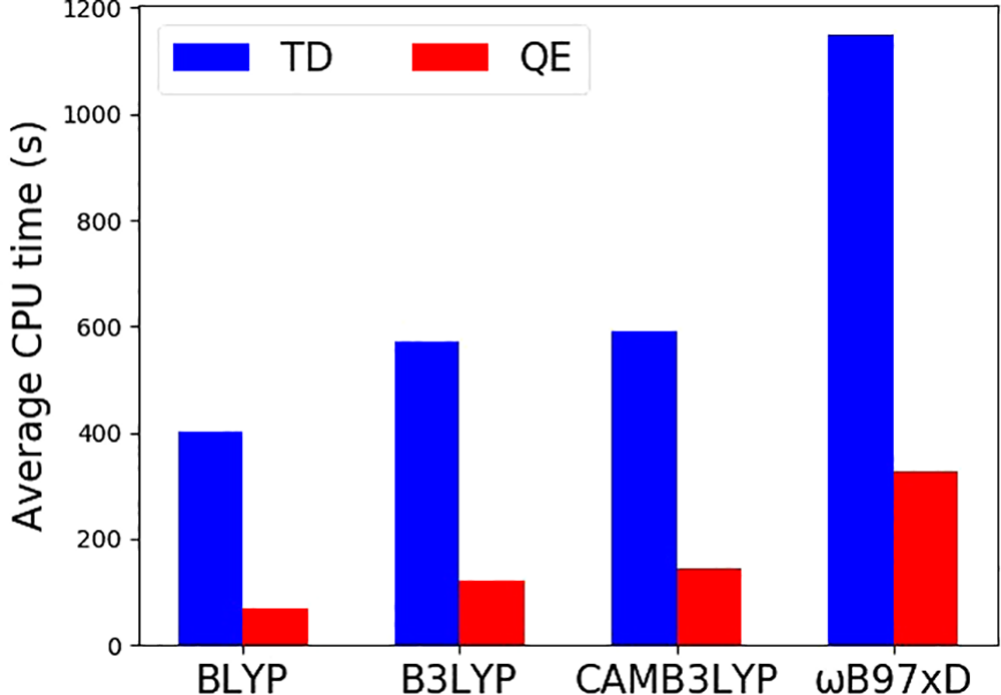
Average total CPU time using TD (blue) and QE (red) calculations from different functionals. All basis sets are 6-31+G*.

One advantage of calculating coupling from QE approaches is the reduction of calculation time. Figure 10 compares the average CPU time using the TD and QE methods. QE requires significantly less CPU time than TD. Therefore, the QE method has a potential advantage of predicting ET couplings for large systems or when embedded in dynamical simulation, which we will investigate in the future.

### On the Effects of Spin Purification

Excitation energies from QE are often improved with spin purification treatments (eq 24), which is also seen in Figure 3. However, in the present implementation, for GMH and FCD, spin purification is not included. Both GMH and FCD require transforms of the Hamiltonian and a property matrix (dipole or charge difference) that depends on the wave functions of states in the model. Thus, an improvement of the excitation energy (which changes the Hamiltonian) would also require improvement in the wave functions and their corresponding properties, such that a sensible calculation for the coupling can be performed. At present, a spin-purified description for the QE transition has yet to be developed. Therefore, we report QE coupling values without spin purification, including couplings from an electric field scan (Figure 9), offering valuable, low-cost alternatives to TD-DFT for excited state coupling.

In this study, we demonstrated that energies and electronic coupling can be calculated from QE methods. QE methods are useful in obtaining excitation energies. Furthermore, (LOSC-SCF corrected) QE couplings are also accurate compared with conventional GMH and FCD schemes from TDDFT. In addition, coupling values calculated by two-state methods are consistent with those estimated by the electric field approach. Considering the accuracy and the relatively inexpensive calculation, we believe that this method provides an efficient way to describe CT couplings, especially for large systems, which will be the focus of future studies.

## Supporting Information Available

Description of technical details of excitation energies, absolute value of couplings, FCD couplings using Löwdin population, and additional figures (PDF)
