# Supplementary material for: Excited-State Charge Transfer Coupling from Quasiparticle Energy Density Functional Theory
Source: J Phys Chem Lett. 2024 Jun 3;15(23):6126–36. doi: 10.1021/acs.jpclett.4c00850 (PMC11181311; doi:10.1021/acs.jpclett.4c00850)
Supplement: Supplementary file 1 — jz4c00850_si_001.pdf [file jz4c00850_si_001.pdf]

Supporting Information: Excited-State Charge Transfer Coupling from Quasiparticle Energy Density Functional Theory

Kai-Yuan Kuan,[†] Shu-Hao Yeh,[†,‡] Weitao Yang,[*,¶] and Chao-Ping Hsu[*,†,§]

†*128 Academia Road, Section 2, Nankang, Taipei 11529, Taiwan*

‡*Department of Chemistry, National Taiwan University, 1, Section 4, Roosevelt Rd, Da'an District, Taipei City, 10617*

¶*Duke University, Durham, North Carolina 27708, United States*

§*Division of Phyiscs, National Center for Theoretical Sciences, 1, Section 4, Roosevelt Road, Taipei 106, Taiwan*

E-mail: weitao.yang@duke.edu; cherri@sinica.edu.tw

Excitation energies

In this work, the excitation energies of the four states (LE_S, LE_A, CT_S, and CT_A) can be calculated from two different approaches: the conventional TDDFT and the QE approaches. For the QE approach, a simple approximation can be made by taking orbital energy difference. Alternatively, one could apply the spin-purification process. Both are described more detailed in the main text. We refer the former scheme to "orbital difference," and the latter one to "spin-purification" in the following discussion. The spin-purification corrections becomes significant when the energy difference between an α-orbital and the corresponding β-orbital is large. Energies calculated from QE approach can be further corrected using previous developed localized-orbital scaling correction (LOSC-SCF) method.[1] The excitation energies using these approaches for the four states are summarized in the following tables of this section, followed by plots showing the trends of these states. All the basis sets are 6-31+G* unless mentioned otherwise.

An attempt was made to compare excitation energies with high-level *ab initio* methods. The computational costs of such complex is marginally prohibitory for a standard EOM-CCSD(T) calculation. However, a much improved approach using similarity transformed equation-of-motion domain-based pair natural orbital coupled cluster single and double (STEOM-DLPNO-CCSD) method can greatly reduce the computational time while maintaining the couple-cluster accuracy.[2] However, we note that calculation using the STEOM-DLPNO-CCSD/aug-cc-pVDZ method did not afford unambiguous states that can be assigned as one of the four states discussed in this work. Table S1 summarizes the results in this calculation. Despite the issues, excitation energies at the d = 3.5 Å indicate that the excitation energies for both TD and QE methods are slightly underestimated. However, the LOSC-SCF on BLYP, B3LYP, and CAMB3LYP show a significant correction toward the STEOM-DLPNO-CCSD results with the LOSCSCF-CAMB3LYP one having the smallest displacement. This indicates that the LOSC-SCF approach successfully correct excitation energies with CT characters from those in the TDDFT.[3] Meanwhile, our earlier work shows

that dynamical correlation does not affect charge-transfer coupling much.[4] Therefore, the level of calculation in the present work, with well-characterized DFT, should be sufficient already.

Crude excitation energies

TD-BLYP

	TD-BLYP			
d (Å)	LE_A	LE_S	CT_A	CT_S
3.5	5.154	5.457	2.311	3.580
4.0	5.126	5.421	2.134	3.355
4.5	5.111	5.414	2.031	3.233
5.0	5.104	5.407	1.964	3.158
5.5	5.098	5.401	1.913	3.103
6.0	5.096	5.399	1.879	3.067

QE-BLYP

QE-BLYP	orbital difference				spin-purification			
d (Å)	LE_A	LE_S	CT_A	CT_S	LE_A	LE_S	CT_A	CT_S
3.5	5.270	3.811	4.075	5.292	5.308	4.341	4.096	5.310
4.0	5.257	4.616	4.880	6.228	5.295	4.356	4.299	5.456
4.5	5.248	3.820	4.285	5.468	5.286	4.354	4.460	5.640
5.0	5.242	4.591	5.241	6.580	5.279	4.351	4.592	5.787
5.5	5.239	3.820	4.446	5.635	5.276	4.349	4.686	5.879
6.0	5.236	4.622	5.577	6.915	5.272	4.347	4.746	5.910

QE-BLYP LOSCSCF

QE-BLYP LOSCSCF	orbital difference				spin-purification			
d (Å)	LE_A	LE_S	CT_A	CT_S	LE_A	LE_S	CT_A	CT_S
3.5	7.183	4.587	4.590	5.964	7.228	5.369	4.614	5.987
4.0	7.188	4.616	4.880	6.228	7.233	5.410	4.898	6.241
4.5	7.163	4.600	5.071	6.413	7.209	5.396	5.080	6.418
5.0	7.146	4.591	5.241	6.580	7.188	5.387	5.247	6.585
5.5	7.161	4.609	5.424	6.761	7.206	5.405	5.433	6.767
6.0	7.172	4.622	5.577	6.915	7.217	5.419	5.585	6.920

TD-B3LYP

	TD-B3LYP			
d (Å)	LE_A	LE_S	CT_A	CT_S
3.5	5.719	5.970	3.086	4.480
4.0	5.688	5.952	3.001	4.371
4.5	5.672	5.957	2.965	4.324
5.0	5.664	5.950	2.952	4.306
5.5	5.658	5.944	2.948	4.298
6.0	5.655	5.950	2.951	4.299

QE-B3LYP

QE-B3LYP	orbital difference				spin-purification			
d (Å)	LE_A	LE_S	CT_A	CT_S	LE_A	LE_S	CT_A	CT_S
3.5	5.685	4.212	4.351	5.738	5.729	5.077	4.365	5.764
4.0	5.662	4.220	4.573	5.916	5.706	5.096	4.579	5.897
4.5	5.652	4.219	4.776	6.130	5.696	5.096	4.779	6.133
5.0	5.648	4.218	4.959	6.310	5.692	5.095	4.960	6.311
5.5	5.645	4.217	5.127	6.476	5.690	5.094	5.128	6.510
6.0	5.644	4.217	5.273	6.621	5.688	5.093	5.273	6.640

QE-B3LYP LOSCSCF

QE-B3LYP LOSCSCF	orbital difference				spin-purification			
d (Å)	LE_A	LE_S	CT_A	CT_S	LE_A	LE_S	CT_A	CT_S
3.5	7.573	4.771	4.977	6.318	7.621	6.092	4.793	6.353
4.0	7.573	5.006	5.030	6.541	7.621	6.137	5.048	6.557
4.5	7.549	4.993	5.219	6.721	7.598	6.125	5.227	6.728
5.0	7.533	4.985	5.391	6.891	7.579	6.116	5.398	6.896
5.5	7.549	5.004	5.578	7.075	7.598	6.135	5.585	7.079
6.0	7.560	5.017	5.733	7.230	7.608	6.149	5.741	7.235

TD-CAMB3LYP

	TD-CAMB3LYP			
d (Å)	LE_A	LE_S	CT_A	CT_S
3.5	6.256	6.282	4.204	5.613
4.0	6.224	6.226	4.295	5.730
4.5	6.204	6.210	4.404	5.847
5.0	6.199	6.202	4.513	5.958
5.5	6.196	6.198	4.614	6.058
6.0	6.193	6.198	4.707	6.150

QE-CAMB3LYP

QE-CAMB3LYP	orbital difference				spin-purification			
d (Å)	LE_A	LE_S	CT_A	CT_S	LE_A	LE_S	CT_A	CT_S
3.5	7.102	4.574	4.584	6.084	6.138	5.715	4.596	6.123
4.0	6.076	4.589	4.802	7.085	6.123	5.745	4.807	6.287
4.5	6.068	4.590	5.006	6.460	6.115	5.748	5.009	6.463
5.0	6.065	4.590	5.191	6.640	6.112	5.747	5.193	6.640
5.5	6.063	4.590	5.358	6.804	6.110	5.747	5.359	6.803
6.0	6.062	4.590	5.503	6.948	6.109	5.747	5.505	6.947

QE-CAMB3LYP LOSCSCF

QE-CAMB3LYP	orbital difference				spin-purification			
d (Å)	LE_A	LE_S	CT_A	CT_S	LE_A	LE_S	CT_A	CT_S
3.5	7.697	5.159	4.987	6.609	7.749	6.511	4.997	6.649
4.0	7.674	5.166	5.198	6.780	7.759	6.533	5.198	6.788
4.5	7.665	5.163	5.400	6.971	7.754	6.532	5.402	6.975
5.0	7.665	5.165	5.589	7.156	7.756	6.534	5.585	7.153
5.5	7.668	5.169	5.758	7.323	7.759	6.537	5.759	7.324
6.0	7.661	5.162	5.899	7.462	7.753	6.529	5.899	7.463

TD-ωB97xD

	TD-ωB97xD			
d (Å)	LE_A	LE_S	CT_A	CT_S
3.5	6.233	6.298	4.235	5.645
4.0	6.206	6.240	4.387	5.818
4.5	6.191	6.227	4.565	6.003
5.0	6.182	6.233	4.742	6.170
5.5	6.176	6.210	4.906	6.358
6.0	6.172	6.209	5.058	6.507

QE-ωB97xD

QE-ωB97xD	orbital difference				spin-purification			
d (Å)	LE_A	LE_S	CT_A	CT_S	LE_A	LE_S	CT_A	CT_S
3.5	6.095	4.574	4.613	6.115	6.147	5.670	4.625	6.147
4.0	6.077	4.589	4.830	6.293	6.129	5.699	4.834	6.347
4.5	6.069	4.591	5.037	6.496	6.121	5.704	5.039	6.497
5.0	6.065	4.591	5.223	6.678	6.116	5.704	5.225	6.678
5.5	6.062	4.591	5.387	6.839	6.114	5.703	5.388	6.839
6.0	6.060	4.591	5.533	6.984	6.112	5.702	5.534	7.030

EOM-CCSD/aug-cc-pVDZ

Table S1: STEOM-DLPNO-CCSD/aug-cc-pVDZ results.

	EOMCCSD/aug-cc-pVDZ			
d (Å)	LE_A	LE_S	CT_A	CT_S
3.5	7.109	6.758	5.119	6.07
4.0	^{a}	^{a}	^{a}	^{a}
4.5	^{a}	^{a}	5.582	^{a}
5.0	^{a}	^{a}	5.716, 5.733^b	^{a}
5.5	^{a}	^{a}	5.826, 5.839^b	^{a}
6.0	^{a}	^{a}	5.893	^{a}

^{a} state not converged.
^{b} existing of two states with similar amplitudes at the desired transition (f > 0.8), but with symmetric and antisymmetric mixing with another transition.

Plots of excitation energies

BLYP

(a) (b) (c)

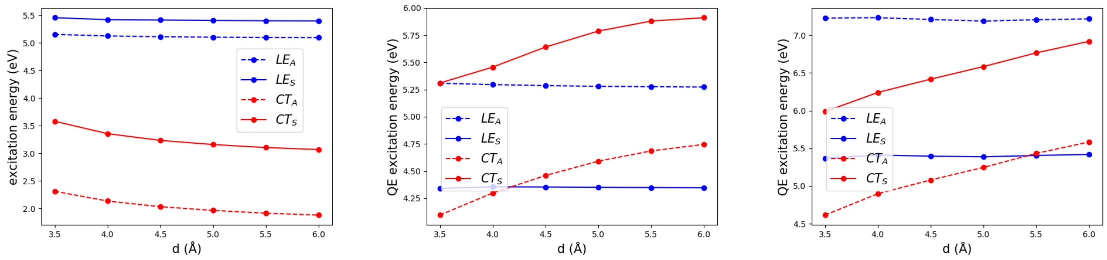

Figure S1: Excitation energies with spin purification calculated by BLYP functional from (a) TD, (b) QE, and (c) QE with LOSC-SCF correction.

B3LYP

(a) (b) (c)

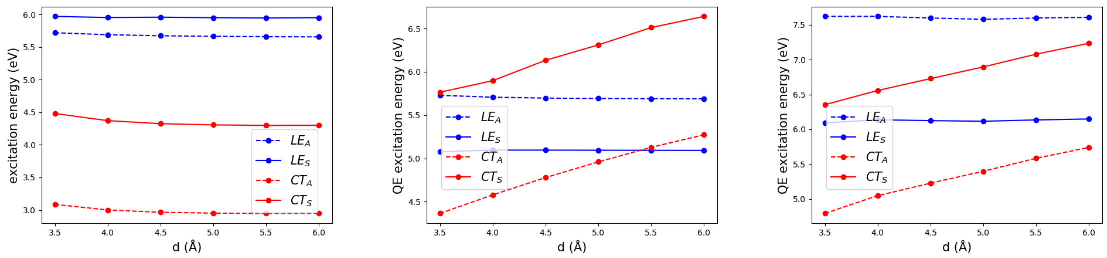

Figure S2: Excitation energies calculated by B3LYP functional from (a) TD, (b) QE, and (c) QE with LOSC-SCF correction.

CAMB3LYP

(a) (b) (c)

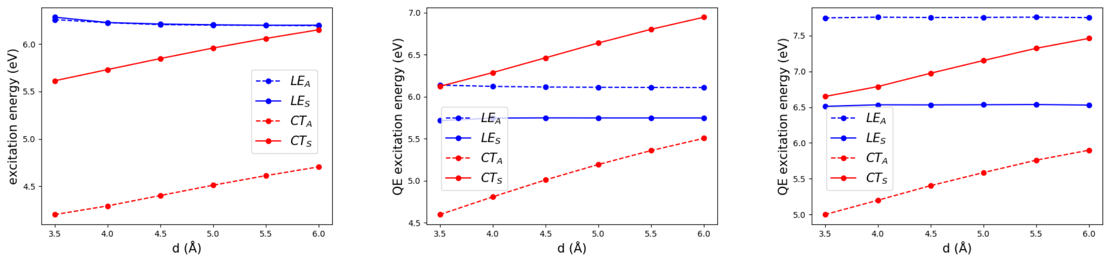

Figure S3: Excitation energies calculated by CAMB3LYP functional from (a) TD, (b) QE approaches, and (c) QE with LOSC-SCF correction.

ωB97xD

(a) (b)

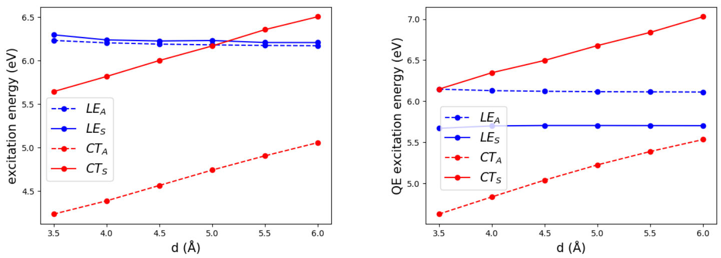

Figure S4: Excitation energies calculated by ωB97xD functional from (a) TD, and (b) QE approach.

GMH and FCD Couplings

This section summarizes crude values of couplings using GMH and FCD schemes. For the FCD couplings, the integration expressed by Eq. (20) in the main text can be taken via Mülliken population, and the difference between donor and acceptor fragments for $i = j$, and is one-particle transition density for $m \neq n$. For a transition density, Δq derived from the the Mülliken population is not symmetric. Therefore, the averaged values ($\Delta q_{ij}^{\text{avg}} = (\Delta q_{ij} + \Delta q_{ji})/2)$) were taken for the off-diagonal Δq_{ij} in calculating FCD couplings. Alternatively, since the trace of population matrices is unchanged upon transformation with any α:

$$tr(\mathbf{P}) = tr(\mathbf{DS}) = tr(\mathbf{S^{1-\alpha}}\mathbf{DS}^{\alpha}), \tag{1}$$

one could use the Löwdin population[5] by taking $\alpha = 0.5$:

$$\mathbf{P}_{ij}^{L} = \mathbf{S}^{1/2}\mathbf{D}_{ij}\mathbf{S}^{1/2}. \tag{2}$$

We denote FCD-M, and FCD-L as the FCD couplings using Mülliken and Löwdin population, respectively.

GMH couplings

$\mathbf{LE}_S$ – $\mathbf{CT}_S$

method	functional	3.5	4.0	4.5	5.0	5.5	6.0
TD	BLYP	0.1980	0.1020	0.0530	0.0260	0.0130	0.0062
	B3LYP	0.2100	0.1100	0.0570	0.0280	0.0130	0.0064
	CAMB3LYP	0.2320	0.1230	0.0650	0.0330	0.0170	0.0083
	ωB97xD	0.2287	0.1166	0.0589	0.0303	0.0143	0.0078
QE	BLYP	0.1978	0.0962	0.0570	0.0285	0.0141	0.0069
	B3LYP	0.2283	0.1245	0.0645	0.0322	0.0160	0.0080
	CAMB3LYP	0.2632	0.1360	0.0768	0.0389	0.0195	0.0098
	ωB97xD	0.2619	0.1350	0.0763	0.0383	0.0195	0.0101
QE-LOSCSCF	BLYP	0.2738	0.1091	0.0569	0.0288	0.0143	0.0070
	B3LYP	0.2340	0.1242	0.0638	0.0320	0.0158	0.0077
	CAMB3LYP	0.2710	0.1463	0.0765	0.0389	0.0194	0.0096

$\mathbf{LE}_A$ – $\mathbf{CT}_A$

method	functional	3.5	4.0	4.5	5.0	5.5	6.0
TD	BLYP	0.03257	0.01511	0.00876	0.00521	0.00169	0.00073
	B3LYP	0.03062	0.01397	0.00470	0.01005	0.00358	0.00127
	CAMB3LYP	0.00828	0.01575	0.00845	0.00407	0.00189	0.00085
	ωB97xD	0.03355	0.01573	0.00701	0.00312	0.00131	0.00056
QE	BLYP	0.02540	0.01245	0.00586	0.00268	0.00123	0.00060
	B3LYP	0.02816	0.01388	0.00656	0.00300	0.00136	0.00063
	CAMB3LYP	0.03236	0.01620	0.00772	0.00355	0.00159	0.00071
	ωB97xD	0.03327	0.01630	0.00766	0.00354	0.00162	0.00072
QE-LOSCSCF	BLYP	0.03394	0.01255	0.00598	0.00273	0.00128	0.00060
	B3LYP	0.02872	0.01422	0.00671	0.00303	0.00138	0.00061
	CAMB3LYP	0.03248	0.01641	0.00783	0.00360	0.00160	0.00068

FCD couplings (Mülliken population)

LE$_S$ – CT$_S$

method	functional	3.5	4.0	4.5	5.0	5.5	6.0
TD	BLYP	0.1820	0.0910	0.0460	0.0220	0.0100	0.0045
	B3LYP	0.2050	0.1050	0.0540	0.0260	0.0120	0.0056
	CAMB3LYP	0.2300	0.1220	0.0650	0.0330	0.0170	0.0083
	ωB97xD	0.2278	0.1163	0.0589	0.0303	0.0144	0.0078
QE	BLYP	0.1835	0.1128	0.0517	0.0267	0.0134	0.0067
	B3LYP	0.2128	0.1415	0.0597	0.0309	0.0157	0.0079
	CAMB3LYP	0.2473	0.1354	0.0720	0.0375	0.0192	0.0098
	ωB97xD	0.2475	0.1406	0.0718	0.0372	0.0195	0.0102
QE-LOSCSCF	BLYP	0.2063	0.0979	0.0527	0.0275	0.0141	0.0070
	B3LYP	0.2091	0.1113	0.0592	0.0306	0.0154	0.0077
	CAMB3LYP	0.2710	0.1463	0.0765	0.0389	0.0194	0.0096

LE$_A$ – CT$_A$

method	functional	3.5	4.0	4.5	5.0	5.5	6.0
TD	BLYP	0.03099	0.01643	0.00972	0.00521	0.00272	0.00139
	B3LYP	0.03139	0.01624	0.00784	0.00782	0.00335	0.00148
	CAMB3LYP	0.02035	0.01618	0.00884	0.00455	0.00225	0.00108
	ωB97xD	0.03272	0.01608	0.00761	0.00359	0.00160	0.00071
QE	BLYP	0.02376	0.01228	0.00614	0.00297	0.00143	0.00073
	B3LYP	0.02659	0.01373	0.00682	0.00324	0.00150	0.00070
	CAMB3LYP	0.03024	0.01582	0.00793	0.00381	0.00177	0.00081
	ωB97xD	0.03111	0.01572	0.00766	0.00366	0.00171	0.00078
QE-LOSCSCF	BLYP	0.02623	0.01321	0.00729	0.00383	0.00201	0.00103
	B3LYP	0.02696	0.01485	0.00792	0.00407	0.00209	0.00103
	CAMB3LYP	0.03024	0.01670	0.00884	0.00453	0.00224	0.00107

FCD couplings (Löwdin population)

LE_S – CT_S

method	functional	3.5	4.0	4.5	5.0	5.5	6.0
QE	BLYP	0.1785	0.1081	0.0466	0.0228	0.0107	0.0048
	B3LYP	0.2070	0.1360	0.0537	0.0262	0.0123	0.0056
	CAMB3LYP	0.2406	0.1284	0.0653	0.0325	0.0157	0.0073
	ωB97xD	0.2405	0.1334	0.0650	0.0321	0.0158	0.0077
QE-LOSCSCF	BLYP	0.2738	0.1091	0.0569	0.0288	0.0143	0.0070
	B3LYP	0.2037	0.1053	0.0537	0.0263	0.0124	0.0056
	CAMB3LYP	0.2397	0.1263	0.0654	0.0326	0.0157	0.0073

LE_A – CT_A

method	functional	3.5	4.0	4.5	5.0	5.5	6.0
QE	BLYP	0.01990	0.00997	0.00477	0.00222	0.00105	0.00053
	B3LYP	0.02215	0.01115	0.00537	0.00251	0.00117	0.00056
	CAMB3LYP	0.02535	0.01293	0.00626	0.00292	0.00134	0.00061
	ωB97xD	0.02648	0.01318	0.00629	0.00295	0.00138	0.00063
QE-LOSCSCF	BLYP	0.01677	0.00769	0.00361	0.00160	0.00076	0.00037
	B3LYP	0.01789	0.00893	0.00408	0.00175	0.00078	0.00033
	CAMB3LYP	0.02158	0.01109	0.00524	0.00237	0.00102	0.00042

Couplings from Electric Field Methods

The electric field methods for calculating electronic coupling was performed by searching for the minimum of the energy gap between two states of interests. This was achieved by scanning the external electric field strength in a range covering the minimum gap. The scan was repeated with a narrower increment until the orbital energies of the HOMO-2 and HOMO-3 at its minimum gap and the two adjacent points are consistent at least three significant figures. The following table summarizes the results of the final scan in the neighborhood of the minimum point. The negative field corresponds to the LE_A – CT_A coupling and the positive charge corresponds to the LE_S – CT_S. The gap energy represents the energy difference (eV)

of HOMO-2 and HOMO-3 orbitals , which correspond to the two crossing hole orbitals in 1-electron picture of LE and CT.

d = 3.5 Å

	F (a.u.)	HOMO-2	HOMO-3	Gap (eV)
	-0.0368	-0.1876	-0.1950	0.2020
	-0.0364	-0.1879	-0.1952	0.2006
$LE_A - CT_A$	-0.0360	-0.1881	-0.1955	0.2005
	-0.0356	-0.1884	-0.1958	0.2015
	-0.0352	-0.1886	-0.1961	0.2037
	0.0336	-0.2506	-0.2678	0.4695
	0.0340	-0.2510	-0.2682	0.4688
$LE_S - CT_S$	0.0344	-0.2514	-0.2687	0.4688
	0.0348	-0.2518	-0.2691	0.4695
	0.0352	-0.2522	-0.2696	0.4708

d = 4.0 Å

	F (a.u.)	HOMO-2	HOMO-3	Gap (eV)
	-0.0257	-0.2012	-0.2024	0.03350
	-0.0256	-0.2013	-0.2025	0.03247
$LE_A - CT_A$	-0.0255	-0.2014	-0.2026	0.03196
	-0.0254	-0.2016	-0.2027	0.03202
	-0.0253	-0.2016	-0.2028	0.03265
	0.0320	-0.2603	-0.2696	0.2522
	0.0324	-0.2608	-0.2700	0.2497
$LE_S - CT_S$	0.0328	-0.2613	-0.2705	0.2489
	0.0332	-0.2618	-0.2710	0.2499
	0.0336	-0.2622	-0.2715	0.2526

d = 4.5 Å

	F (a.u.)	HOMO-2	HOMO-3	Gap (eV)
	-0.017720	-0.21162	-0.21217	0.014953
	-0.017716	-0.21163	-0.21218	0.014948
$LE_A - CT_A$	-0.017712	-0.21163	-0.21218	0.014946
	-0.017708	-0.21164	-0.21219	0.014948
	-0.017704	-0.21164	-0.21219	0.014953
	0.0308	-0.2686	-0.2737	0.1390
	0.0312	-0.2693	-0.2741	0.1312
$LE_S - CT_S$	0.0316	-0.2698	-0.2745	0.1275
	0.0320	-0.2703	-0.2751	0.1285
	0.0324	-0.2708	-0.2757	0.1339

d = 5.0 Å

	F (a.u.)	HOMO-2	HOMO-3	Gap (eV)
	-0.012652	-0.219682	-0.219936	0.006908
	-0.012648	-0.219688	-0.219941	0.006895
$LE_A - CT_A$	-0.012644	-0.219693	-0.219946	0.006892
	-0.012640	-0.219698	-0.219952	0.006900
	-0.012636	-0.219703	-0.219957	0.006917
	0.0300	-0.2759	-0.2789	0.08086
	0.0304	-0.2767	-0.2792	0.06756
$LE_S - CT_S$	0.0308	-0.2773	-0.2797	0.06399
	0.0312	-0.2778	-0.2804	0.07165
	0.0316	-0.2780	-0.2812	0.08763

d = 5.5 Å

	F (a.u.)	HOMO-2	HOMO-3	Gap (eV)
	-0.009072	-0.226445	-0.226564	0.003215
	-0.009068	-0.226452	-0.226569	0.003177
$LE_A - CT_A$	-0.009064	-0.226458	-0.226574	0.003165
	-0.009060	-0.226463	-0.226580	0.003181
	-0.009056	-0.226468	-0.226587	0.003224
	0.02968	-0.226445	-0.226564	0.03226
	0.02970	-0.226452	-0.226569	0.03218
$LE_S - CT_S$	0.02972	-0.226458	-0.226574	0.03217
	0.02974	-0.226463	-0.226580	0.03223
	0.02976	-0.226468	-0.226587	0.03237

d = 6.0 Å

	F (a.u.)	HOMO-2	HOMO-3	Gap (eV)
	-0.0064090	-0.2323025	-0.2323546	0.00141706
	-0.0064088	-0.2323028	-0.2323549	0.00141671
$LE_A - CT_A$	-0.0064086	-0.2323032	-0.2323552	0.00141654
	-0.0064084	-0.2323035	-0.2323555	0.00141654
	-0.0064082	-0.2323038	-0.2323559	0.00141676
	0.02866	-0.28842	-0.28903	0.01676
	0.02868	-0.28846	-0.28906	0.01644
$LE_S - CT_S$	0.02870	-0.28849	-0.28909	0.01628
	0.02872	-0.28852	-0.28912	0.01630
	0.02874	-0.28855	-0.28916	0.01649

References

(1) Mei, Y.; Chen, Z.; Yang, W. Self-Consistent Calculation of the Localized Orbital Scaling Correction for Correct Electron Densities and Energy-Level Alignments in Density Functional Theory. *J. Phys. Chem. Lett.* **2020**, *11*, 10269–10277.

(2) Izsák, R. A Local Similarity Transformed Equation of Motion Approach for Calculating Excited States. *Int. J. Quantum Chem.* **2021**, *121*, e26327.

(3) Yang, Y.; Dominguez, A.; Zhang, D.; Lutsker, V.; Niehaus, T. A.; Frauenheim, T.; Yang, W. Charge Transfer Excitations from Particle-Particle Random Phase Approximation-Opportunities and Challenges Arising from Two-Electron Deficient Systems. *J. Chem. Phys.* **2017**, *146*, 124104.

(4) Yang, C.-H.; Hsu, C.-P. The Dynamical Correlation in Spacer-Mediated Electron Transfer Couplings. *J. Chem. Phys.* **2006**, *124*, 244507.

(5) Löwdin, P.-O. On the Non-Orthogonality Problem Connected with the Use of Atomic Wave Functions in the Theory of Molecules and Crystals. *J. Chem. Phys.* **2004**, *18*, 365–375.
